# Thinking Outside the Box: Indirect Myc Modulation in Canine B-Cell Lymphoma

**DOI:** 10.3390/ani14101466

**Published:** 2024-05-15

**Authors:** Luca Licenziato, Eugenio Mazzone, Chiara Tarantelli, Paolo Accornero, Andrea Rinaldi, Sara Divari, Wilfred Leung, Suzin Webb, Raffaella De Maria, Luca Aresu

**Affiliations:** 1 Department of Veterinary Sciences, University of Turin, 10095 Grugliasco, Italy; luca.licenziato@unito.it (L.L.); eugenio.mazzone@unito.it (E.M.); paolo.accornero@unito.it (P.A.); sara.divari@unito.it (S.D.); raffaella.demaria@unito.it (R.D.M.); 2 Institute of Oncology Research, Faculty of Biomedical Sciences, USI, 6500 Bellinzona, Switzerland; chiara.tarantelli@ior.usi.ch (C.T.); andrea.rinaldi@ior.usi.ch (A.R.); 3 Department of Biomedical Sciences, College of Veterinary Medicine, Cornell University, Ithaca, NY 14853, USA; wl475@cornell.edu; 4 Velocity Clinical Research, Binghamton, NY 13905, USA; smw266@cornell.edu

**Keywords:** canine B-cell lymphoma, targeted therapy, Myc, BI2536, MZ1

## Abstract

**Simple Summary:**

B-cell lymphoma (BCL) represents the most common hematological malignancy in dogs and, despite the current chemotherapeutic standard, is generally characterized by poor outcome, highlighting the importance of new drug development. The medical approach to cancer has been revolutionized by the advent of targeted therapies, consisting in targeting specific molecules crucial for malignant cells growth and proliferation. Myc is a transcription factor dysregulated in many cancers, including canine BCL (cBCL). For several reasons, directly targeting Myc remains challenging, but alternative routes have been explored. In our study, we developed and tested on two in vitro models of cBCL a dual approach to indirectly target Myc using two drugs (BI2536 and MZ1). After confirming the specificity of the drugs to their main targets, we observed that both molecules affected the cell viability, individually and in combination. Furthermore, BI2536, alone and in combination with MZ1, induced significant transcriptomic changes in cBCL cell lines, primarily affecting *MYC* target genes and genes involved in the cell cycle regulation. Our study offers valuable insights into the mechanisms of action of BI2536 and MZ1 in cBCL cell lines and highlights their potential as targeted therapies for this cancer subtype.

**Abstract:**

B-cell lymphomas (BCL) is the most frequent hematological cancer in dogs. Treatment typically consists of chemotherapy, with CHOP-based protocols. However, outcome remains generally poor, urging the exploration of new therapeutic strategies with a targeted approach. Myc transcription factor plays a crucial role in regulating cellular processes, and its dysregulation is implicated in numerous human and canine malignancies, including canine BCL (cBCL). This study aims to evaluate the efficacy of indirectly inhibiting Myc in cBCL using BI2536 and MZ1 compounds in two in vitro models (CLBL-1 and KLR-1201). Both BI2536 and MZ1, alone and combined, affected cell viability in a significant concentration- and time-dependent manner. Western Blot revealed an upregulation of PLK1 expression in both cell lines upon treatment with BI2536, in association with a reduction in c-Myc protein levels. Conversely, MZ1 led to a decrease in its primary target, BRD4, along with a reduction in c-Myc. Furthermore, BI2536, both alone and in combination with MZ1, induced larger transcriptomic changes in cells compared to MZ1 alone, primarily affecting *MYC* target genes and genes involved in cell cycle regulation. These data underscore the potential role of Myc as therapeutic target in cBCL, providing a novel approach to indirectly modulate this molecule.

## 1. Introduction

B-cell lymphomas (BCL) are the most frequent hematological cancer in dogs [1]. Despite ongoing research, the exact cause remains unknown, with environmental factors and genetic predispositions implicated [1]. These tumors present in dogs with heterogenous clinical symptoms and are classified into histological subtypes with varying molecular, epigenetic, and genetic origins [1,2]. Nonetheless, generalized lymphadenopathy is a common feature, often accompanied by varying degrees of blood and bone marrow infiltration [1]. Among BCL histotypes, diffuse large B-cell lymphoma (DLBCL) is predominant, followed by marginal zone lymphoma and follicular lymphoma [3]. Diagnosis primarily relies on histological examination, focusing on recognizing growth pattern and assessing cell size [3]. Immunohistochemical markers such as Pax5, CD79a, and CD20 are routinely employed to confirm the B-cell origin [2,3]. Treatment typically consists of chemotherapy, primarily using a CHOP regimen (cyclophosphamide, doxorubicin, vincristine, prednisone) [4]. However, a notable number of dogs experience tumor relapse, treatment-related toxicity, and short overall survival, necessitating exploration of novel therapeutic strategies with a targeted approach [4,5].

The *MYC* proto-oncogene encodes the Myc transcription factor that is crucial for regulating cellular processes such as proliferation, differentiation, and apoptosis [6]. The dysregulation of Myc is implicated in numerous malignancies in humans, including BCLs [6,7,8]. In canine BCL (cBCL), *MYC* aberrations are also common, with point mutations potentially stabilizing the protein and evading degradation mediated by FBXW7 [9]. Additionally, chromosomal gains affecting whole Chromosome 13 (CFA 13), where *MYC* is located, influence prognosis in canine DLBCL (cDLBCL) [9,10].

Directly targeting Myc poses significant challenges due to its role as a general transcription factor lacking well-defined drug-binding pockets and its predominantly nuclear localization [11]. Alternative strategies that have been proposed are the following: 1. inhibiting *MYC* transcription, 2. targeting *MYC* mRNA translation, 3. modulating its stability, 4. disrupting Myc–Max interaction, and 5. influencing its downstream gene accessibility [11].

Proteolysis-targeting chimeras (PROTACs) are heterobifunctional molecules that degrade specific endogenous proteins, such as oncoproteins, through the E3 ubiquitin ligase pathway [12,13]. PROTACs consist of a target binding unit, a linker, and an E3 ligase binding moiety in which the ubiquitin proteasome system is responsible for degrading the protein of interest [14]. The E3 ligase ligand can hijack the E3 ligase and label the protein with ubiquitin [14]. In this process, PROTACs themselves are not degraded, but are instead recycled to promote the ubiquitination and degradation of other target proteins [14]. In recent years, novel PROTAC agents have been engineered to target proteins more specifically [14]. Of particular interest is the BET degrader MZ1. BET proteins recruit transcription factors involved in an elongation step (P-TEFb) to acetylated chromatin to induce gene transcription and carcinogenesis [15]. MZ1 is derived from JQ1, which exhibits the capacity to degrade BRD2, BRD3, and BRD4 [16]. It demonstrates more potent protein degradation activities compared to JQ1 and shows significant cytotoxicity against colorectal cancer, triple-negative breast cancer, and lymphoma in humans [16,17,18,19].

Another potential candidate drug for targeting Myc is BI2536 [20]. Initially identified as an inhibitor of PLK1, recent studies have demonstrated that BI2536 also interacts with bromodomain functions, potentially resulting in an anti-human lymphoma drug through Myc inactivation [21]. Our hypothesis is that the dual targeting of BI2536 may similarly result in strong antiproliferative effects in canine lymphoma [21]. More importantly, the simultaneous blockade of two targets may reduce the risk of therapy resistance, as the probability of clonal adaptation to targeted therapy is lower for combination therapies [21].

Given these premises, this study aims to evaluate the efficacy of indirectly inhibiting Myc in cBCL using MZ1 and BI2536 compounds in two in vitro models, namely CLBL-1 and KLR-1201. In our experiment, we used the opnMe platform from Boehringer Ingelheim (https://opnme.com, accessed on 14 December 2021) to obtain the two drugs and investigate their anti-proliferative effects in vitro individually and in combination, along with their impacts on c-Myc protein expression and whole transcriptome.

## 2. Materials and Methods

### 2.1. Cell Lines and Molecules

CLBL-1 cells were kindly provided by Dr Barbara Rütgen (University of Wien, Austria). The KLR-1201 cell line was developed in the Drs. Angela McCleary-Wheeler and Kristy Richards’ laboratories (Cornell University, Ithaca, NY, USA). Both cell lines were cultured in IMDM supplemented with fetal bovine serum (10% for CLBL-1; 20% for KLR-1201), 1% glutamine, 100 μg/mL penicillin, and 100 μg/mL streptomycin. BI2536 and MZ1 compounds were purchased from Boehringer Ingelheim (Ingelheim am Rhein, Germany) and were dissolved in dimethyl sulfoxide (DMSO) at 10 µM stock solution.

### 2.2. Cell Proliferation Assay

To evaluate the anti-proliferative effects of the compounds on cBCL cell lines, 15 × 10^4^ cells/well were seeded in 96-well cell culture plates and incubated with BI2536 at 2.5, 5, 7.5, 10, 12.5 and 15nM for 12, 24 and 48 h and with MZ1 at 10, 15, 25, 50, 75 and 100nM for 24, 48 and 72 h, as previously described [22,23]. The effect of the combinations of both drugs was also tested (Table 1). Cells treated with DMSO were used as control. After the treatment, CellTiter 96 AQueous One Solution Cell Proliferation Assay (Promega, Madison, WI, USA) was performed following the manufacturer’s instructions.

### 2.3. Western Blotting

The expression of PLK1, BRD4 and c-Myc proteins was analyzed by Western Blot (WB) in treated and untreated cells. Proteins were extracted in lysis buffer (1% Triton X-100, 10% glycerol, 50mM Tris, 150mM sodium chloride, 2mM EDTA, pH 8.0 and 2mM magnesium chloride) containing protease inhibitor cocktail (Sigma Aldrich, St Louis, MO, USA). Twenty micrograms of total proteins were separated by SDS-PAGE (10% or 15%) and transferred onto a nitrocellulose membrane (Thermo Fisher Scientific, Waltham, MA, USA). After washing, membranes were incubated in TBS/BSA 10% (bovine serum albumin) at room temperature for 1 h and incubated overnight at 4 °C with PLK1 (PA5-95265, Invitrogen, Waltham, MA, USA diluted 1:1000), BRD4 (E2A7X, Cell Signaling Technology, Danvers, MA, USA diluted 1:1000) and c-Myc antibodies (5605T, Cell Signaling Technology, Danvers, MA, USA diluted 1:1000). β-tubulin (T5201, Sigma-Aldrich, St Louis, MO, USA diluted 1:10,000) was used as an internal control. After incubation with horseradish peroxidase (HRP)-linked secondary antibody diluted 1:15,000 in TBS-Tween, membranes were washed 6 times in TBS-Tween and incubated with Clarity Western ECL Substrate (Biorad Laboratories, Hercules, CA, USA). The proteins were visualized by briefly exposing the membrane to an autoradiographic CL-XPosure Film (Thermo Fisher Scientific, Waltham, MA, USA). WB results were then acquired with an Epson scanner.

### 2.4. Transcriptome Profiling

Total RNA was extracted from treated and untreated cells using Maxwell RSC simplyRNA Cells Kit (Promega, Madison, WI, USA) according to the manufacturer’s instructions. RNA concentration and integrity were measured by Nanodrop 2000 Spectrophotometer (ThermoFisher Scientific, Waltham, MA, USA) and assessed through the Bioanalyzer 2010 instrument (Agilent Technologies, Palo Alto, CA, USA). Multiple libraries were prepared for all the conditions described above with the SureSelect Strand Specific RNA-Seq Library Preparation kit (Agilent Technologies, Palo Alto, CA, USA), and a single end sequencing (50SE) was carried out on an Illumina HiSeq2500 (Illumina Inc., San Diego, CA, USA).

All Illumina reads were first analyzed with FastQC software (version 0.12.1) to assess sequence quality, and the resulting quality reports were grouped in a unified report using the MultiQC program. Afterward, the raw data underwent a trimming procedure using Trimmomatic to remove primers and low-quality reads. Briefly, the first and last 20 bp were removed using a sliding window of 4 bp with a required quality score of 20. Additionally, reads shorter than 50 bp were excluded from further analysis. The quality metrics of the data were accessed again after trimming as described above. Finally, the FASTQ files were aligned to the reference genome (canFam3, UCSC) and ENSCAF gene names were annotated with GTF file (UCSC) using STAR aligner. Annotated data were analyzed in R to conduct differential expression analysis using *Limma* and *edgeR* packages. Differentially expressed transcripts were defined as those with an average normalized log expression [counts per million reads mapped (cpm)] of at least 2, presenting an absolute |logFC| ≥ 1 and Benjamini–Hochberg (BH) multiple tests corrected *p*-value [false discovery rate (FDR)] ≤ 0.05. Functional annotation was performed using gene set enrichment analysis (GSEA) on fold-change (FC) pre-ranked lists of genes considered differentially expressed, ranked by the respective logFC and weighted by corresponding adjusted *p*-value. Genesets from the MSigDB collection (hallmark, c4, c6, c7) were used, applying as threshold FDR values ≤ 0.05.

### 2.5. Statistical Analysis

Statistical analyses were performed in R environment. Two-way ANOVA was used to analyze data from proliferation assays to investigate the effects of BI2536 and MZ1 treatment and their combination, and results were considered significant when *p*-value was ≤0.05.

## 3. Results

### 3.1. BI2536 and MZ1 Exhibit Anti-Tumor Activity in Canine B-Cell Lymphoma Cell Lines as Single Agents and in Combination

**Figure 1:**
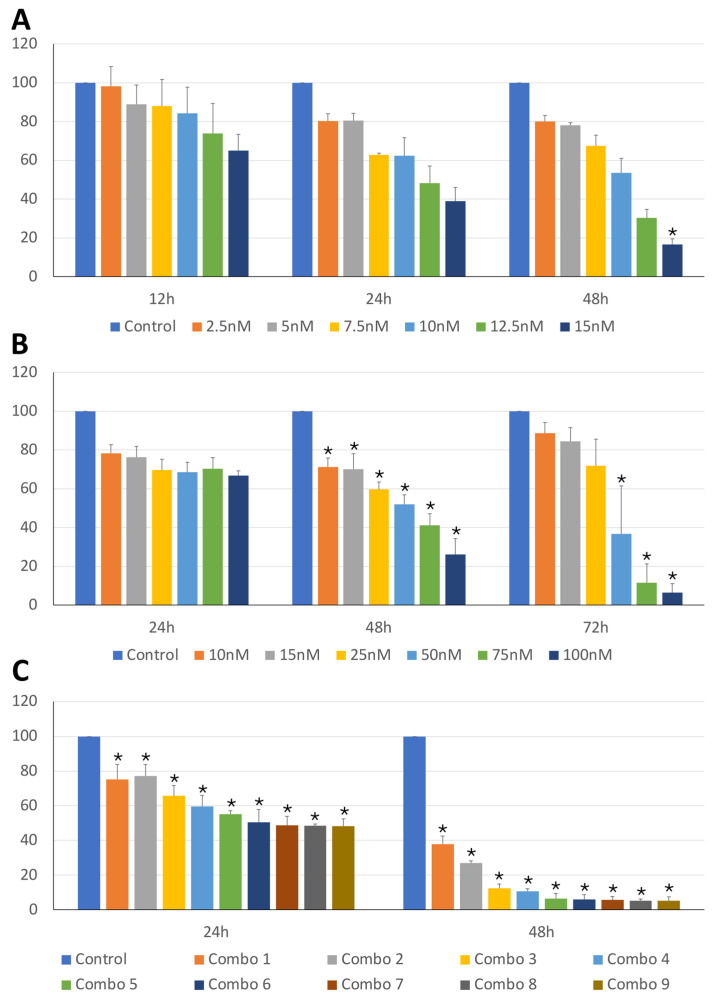
CLBL-1 cell viability under treatment with BI2536 and MZ1, both alone and in combination. Percentage of viable cells (y-axis) in the CLBL-1 cell line treated with BI2536 (**A**), MZ1 (**B**) or combination of both drugs (**C**), at different exposure times and different concentrations, or untreated. The mean value ± SD from at least three independent experiments is illustrated (* *p*-value ≤ 0.05).

**Figure 2:**
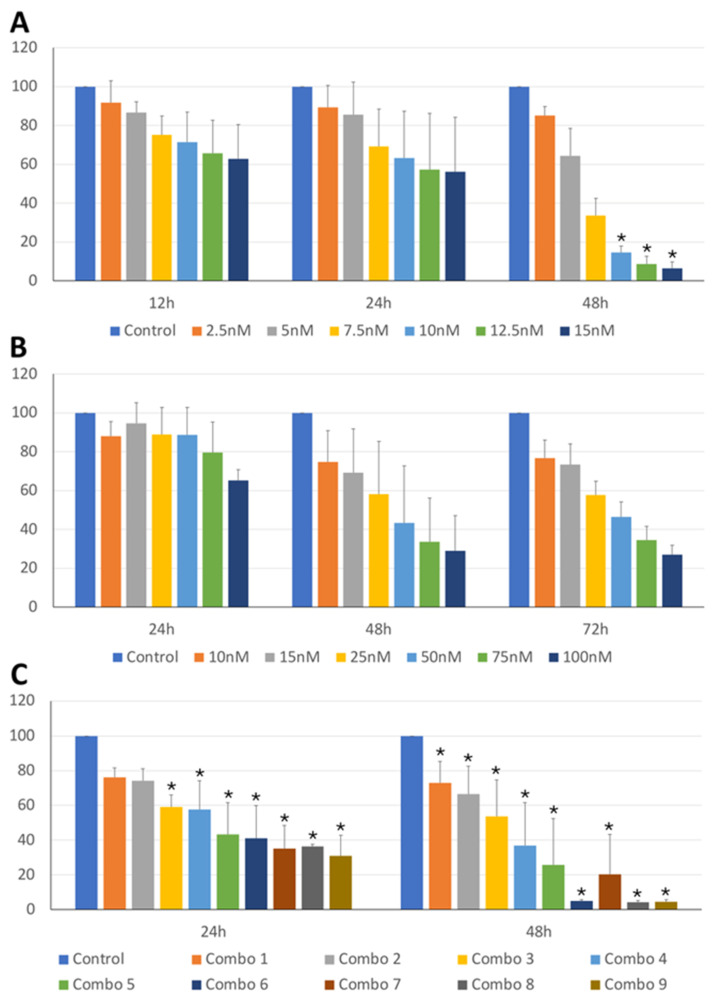
KLR-1201 cell viability under BI2536 and MZ1, both alone and in combination. Percentage of viable cells (y-axis) in the KLR-1201 cell line treated with BI2536 (**A**), MZ1 (**B**) or combination of both drugs (**C**), at different exposure times and different concentrations, or untreated. The mean value ± SD from at least three independent experiments is illustrated (* *p*-value ≤ 0.05).

The anti-tumor activity of BI2536 and MZ1 on cBCL cell lines was assessed. As shown in Figure 1A,B and Figure 2A,B, BI2536 and MZ1 affected the cell viability of CLBL-1 and KLR-1201 cells in a significant concentration- and time-dependent manner (Table S1).

Subsequently, the effects of the combination of the two drugs were also evaluated. The combination led to a concentration-dependent viability reduction in both CLBL-1 and KLR-1201 with a significant time-related effect observed only in KLR-1201 cells (Figure 1C and Figure 2C; Table S1). CLBL-1 cells showed a reduction in cell viability already at low MZ1 doses, with the most dramatic consequences obtained by combining BI2536 15nM with different concentrations of MZ1 (Figure 1C and Figure 2C).

### 3.2. BI2536 and MZ1 Affect c-Myc Protein Expression in Canine B-Cell Lymphoma Cell Lines

**Figure 3:**
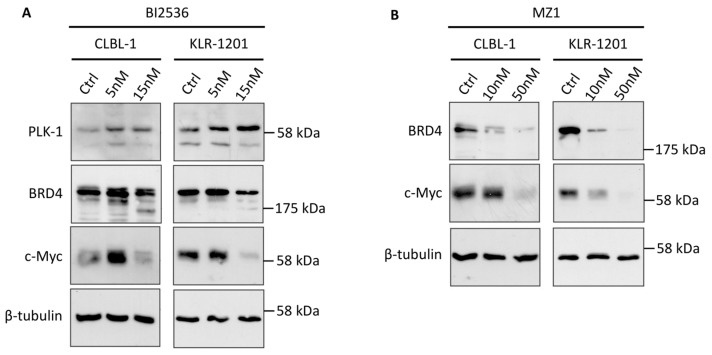
Western Blot (WB) analysis of CLBL-1 and KLR-1201 cells under treatment with BI2536 and MZ1. (**A**) WB analysis of PLK1, BRD4 and c-Myc proteins in CLBL-1 and KLR-1201 under 24 h treatment with BI2536 at 5nM and 15nM. (**B**) WB analysis of BRD4 and c-Myc proteins in CLBL-1 and KLR-1201 under 24 h treatment with MZ1 at 10nM and 50nM.

To evaluate the specificity of BI2536 and MZ1 for their respective targets and their indirect impact on c-Myc expression, WB was conducted on CLBL-1 and KLR-1201 cell lines following a 24 h treatment with concentrations of 5 and 15nM for BI2536 and 10 and 50nM for MZ1, respectively. Remarkably, both cell lines exhibited an upregulation of PLK1 expression when treated with BI2536, in association with a concentration-dependent reduction in c-Myc protein levels, as illustrated in Figure 3A. In line with the dual activity of BI2536, the drug also triggered a decrease in BRD4 expression in both cell lines (Figure 3A). MZ1 demonstrated a conspicuous concentration-dependent decrease in its primary target, BRD4, along with a corresponding reduction in c-Myc protein expression in CLBL-1 and KLR-1201 cells (Figure 3B). Furthermore, the combination of the two drugs induced a downregulation of c-Myc protein expression.

### 3.3. BI2536 Alone and in Combination with MZ1 Induces Transcriptomic Changes in Canine B-Cell Lymphoma Cell Lines

**Figure 4:**
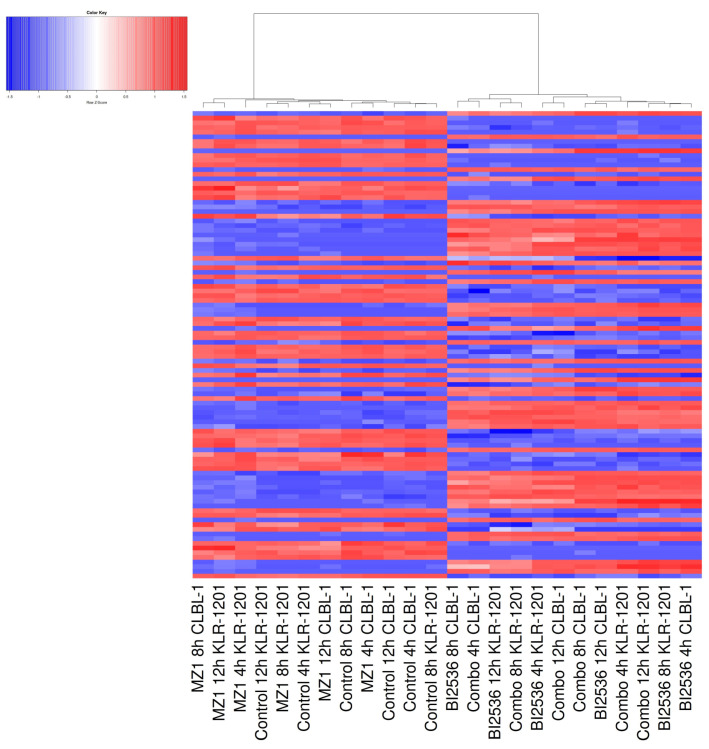
Heatmap showing RNA-seq data obtained from CLBL-1 and KLR-1201 cells under 4, 8 and 12 h treatment with BI2536 and MZ1, alone and in combination. Hierarchical clustering was applied. Data were normalized and scaled via a Z-transformation; red indicates high gene expression; blue indicates low gene expression.

To investigate the impact of both drugs on the whole transcriptome, we conducted RNA-seq analysis after exposing the two BCL cell lines to BI2536 (20nM) and MZ1 (80nM), individually and in combination (BI2536 15nM + MZ1 100nM) for durations of 4, 8, and 12 h. We found significant differences in gene expression only in cells treated with BI2536 and BI2536 and MZ1 combination, but not in cells treated with MZ1 alone (Figure 4; Tables S2 and S3).

**Figure 5:**
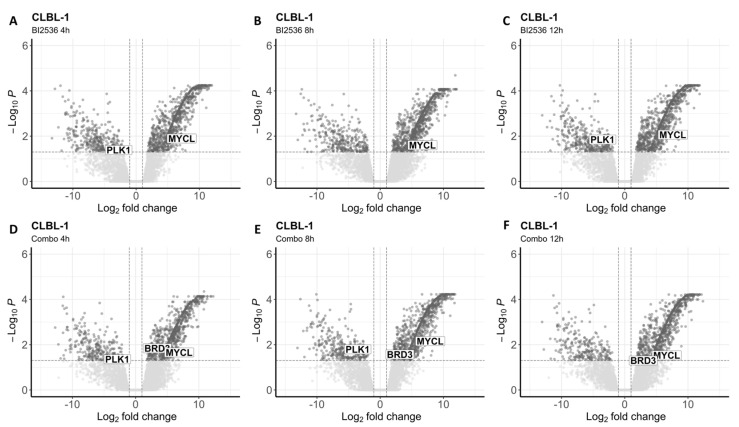
Differentially expressed genes in CLBL-1 after treatment with BI2536 alone or in combination with MZ1. (**A**–**C**) Volcano plots showing significantly upregulated and downregulated transcripts after cells’ exposure to BI2536 at 20nM for 4 (**A**), 8 (**B**), and 12 h (**C**). (**D**–**F**) Volcano plots showing significantly upregulated and downregulated transcripts (*PLK1*, *MYCL* and *BRD3*) after cells’ exposure to BI2536 at 15nM in combination with MZ1 at 100nM for 4 (**D**), 8 (**E**), and 12 h (**F**).

**Figure 6:**
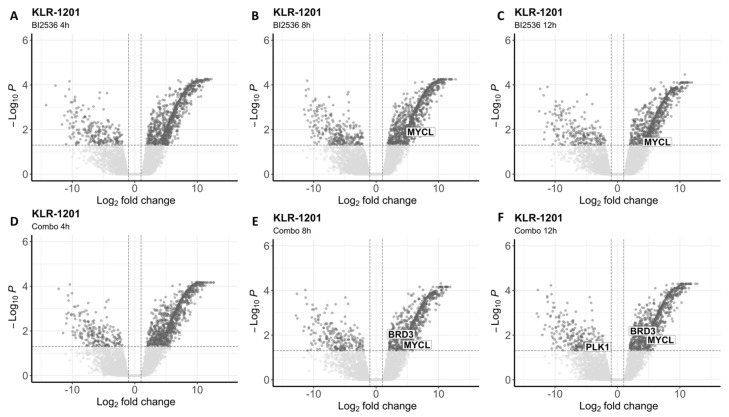
Differentially expressed genes in KLR-1201 after treatment with BI2536 alone or in combination with MZ1. (**A**–**C**) Volcano plots showing significantly upregulated and downregulated transcripts after cells’ exposure to BI2536 at 20nM for 4 (**A**), 8 (**B**), and 12 h (**C**). (**D**–**F**) Volcano plots showing significantly upregulated and downregulated transcripts after cells’ exposure to BI2536 at 15nM in combination with MZ1 at 100nM for 4 (**D**), 8 (**E**), and 12 h (**F**).

The number of differentially expressed transcripts (FDR ≤ 0.05) was similar in CLBL-1 and KLR-1201 when comparing BI2536 to control cell lines (mean = 1231 and 1134, respectively). Across the experiments, an average of 330 and 337 genes were found downregulated, while 902 and 868 were upregulated in CLBL-1 and KLR-1201, respectively (Figure 5A–C and Figure 6A–C). However, the combination of compounds induced a larger change in the transcriptome of the CLBL-1 cell line (mean = 1148) compared to the KLR-1201 cell line (mean = 766). On average, 276 genes were found downregulated and 872 were upregulated in CLBL-1 (Figure 5D–F). Similarly, 174 and 596 genes were down- and upregulated in KLR-1201, respectively (Figure 6D–F). Conversely, when comparing both cell lines treated with the combination of MZ1 and BI2536 with cell lines treated with BI2536 alone, no significant differentially expressed genes were identified.

CLBL-1, BI2536 and MZ1, both alone and in combination, induced a downregulation of PCNA and Ki67-related signatures at each timepoint of exposure (Table S4). Additionally, BI2536 affected the expression of genes regulated by *MYC* and *E2F* as well as those involved in the G2/M checkpoint after 8 and 12 h of incubation (Table S4). Early treatment with MZ1 downregulated *MYC* target genes and mTORC1 signatures, and subsequently, JAK/STAT3 and NF-kB signaling (Table S4). After 8 h of incubation, genes related to *H2AFX* were also downregulated (Table S4). The combination recapitulated the effects observed with the single treatments on the transcriptome (Table S4).

In KLR-1201 cells, BI2536 as a single agent or combined with MZ1 exhibited similar effects as observed in CLBL-1, affecting the same gene signatures (Table S5). Differences were noted in cells treated with MZ1 alone, which induced a downregulation of p53 pathway after 8 h of incubation (Table S5).

Interestingly, we observed the downregulation of *PLK1* in CLBL-1 cells after exposure to BI2536 for 4 or 12 h, and to the combination for 4 or 8 h (Table S2). In the KLR-1201 cell line, downregulation of *PLK1* was observed only after treatment with the drug combination for 12 h (Table S3). In CLBL-1 cells at each time point of exposure and in KLR-1201 cells after 8 and 12 h, the combination of the two drugs did not affect *BRD4* expression but led to the upregulation of *BRD3*, for which MZ1 is known to have a milder affinity (Tables S2 and S3). Among the *MYC* family genes, *MYCL* resulted upregulated in both cell lines treated with BI2536 and in combination (Tables S2 and S3).

## 4. Discussion

In this study, we have developed a dual approach aimed at indirectly targeting Myc using two well-known drugs in human oncology setting [11]. In the first approach we were intrigued by the effects of the dual kinase–bromodomain inhibitor BI2536 in canine lymphoma and we tested the two cBCL cell lines. BI2536 was discovered and developed as a PLK1 kinase inhibitor but was later found to also potently inhibit BRD4 [21]. PLK1 interacts with and phosphorylates FBXW7, leading to its autopolyubiquitination and degradation, thereby promoting Myc stabilization [24]. In a feedforward loop, PLK1 transcription is promoted by stabilized Myc [24]. Therefore, the PLK1/FBXW7/Myc axis creates a positive auto-regulatory signal crucial in promoting tumorigenesis and underscores PLK1 as potential therapeutic target in Myc-overexpressing tumors, as validated in our previous study on osteosarcoma cell lines [23,25]. Here, PLK1 inhibition strongly affected the viability of cBCL cells and resulted in a reduction in c-Myc stabilization, indicated by the decrease in c-Myc protein expression observed in treated cells, and consequently, in the downregulation of *PLK1* gene. Conversely, PLK1 exhibited an increase in protein expression similarly to osteosarcoma cell line models [23]. One plausible explanation for this inconsistency might stem from the interference of Myc-induced *PLK1* transcription due to the intricate interplay of signaling pathways that regulate the cell cycle [23]. On the other hand, BI2536 also affected BRD4 protein expression, in line with its dual inhibitory activity [26].

The indirect effect exerted by PLK1 inhibition on Myc activity was confirmed by the downregulation of *MYC* target genes. However, the expression of *MYC* genes was not significantly affected by the treatment with BI2536, except for *MYCL*, whose upregulation suggests a rebound effect that warrants further investigations. BI2536 strongly affected the cell cycle regulation, compromising *E2F* target genes and genes involved in the G2/M checkpoint. The E2F transcription factor family is involved in the transactivation of target genes crucial for the G1/S transition, and the loss of E2F results in cell cycle arrest [27]. The G2/M checkpoint plays a crucial role in preventing cells with DNA damage from undergoing mitosis. When the G2/M checkpoint fails, cells may undergo erroneous replication and, consequently, apoptosis or cell death [28]. Additionally, two other cell-cycle-related signatures downregulated in cBCL cell lines after BI2536 treatment were associated with PCNA, whose expression is under the control of E2F transcription factors, and Ki67, which is involved in active phases of the cell cycle [29,30].

In the second approach, we tested MZ1, a novel PROTAC-based BRD4 degrader. PROTAC technology has emerged as an effective tool for endogenous protein degradation [14]. MZ1 and other BET-degraders are bifunctional PROTAC molecules consisting of ligands for proteins of interest and covalently linked for E3 ubiquitin ligases, which can suppress tumor growth through ubiquitinate target proteins via the ubiquitin–proteasome system [14]. In our assays, MZ1 specifically degraded BRD4 protein, consequently affecting c-Myc expression and providing a specific target for cBCL. In addition, MZ1 affected the cell viability of CLBL-1 and KLR-1201 cells in a significant concentration- and time-dependent manner. This finding aligns with results obtained with BET degraders in human lymphomas, where BRD4 is selectively degraded by drugs with mechanisms of action involving E3 ligase recruitment to the proteasome [19].

In terms of transcriptome effects, MZ1 exhibited different behavior in the two cBCL cell lines utilized in the study. Specifically, the effects of the drug on CLBL-1 were largely attributed to the interference on *MYC* target genes expression and several signaling pathways (JAK/STAT3, mTORC1, NF-kB) involved in cellular processes such as cell survival and proliferation, which are known to be dysregulated in cancer. Additionally, along with the downregulation of proliferation markers signatures (PCNA and Ki67) as observed after treatment with BI2536, MZ1 also downregulated the signature associated with *H2AFX*. This gene codes for the H2A histone family member X, which undergoes phosphorylation in response to DNA double-strand breaks [31]. Therefore, it appears that the DNA damage response may not be primarily responsible for the cellular modifications induced by MZ1, as observed in KLR-1201 cells, that displayed a downregulation of p53 pathway signature.

Despite these intriguing GSEA results, we did not identify significantly differentially expressed genes in either of the cell lines treated with MZ1. We have ruled out technical issues that could have been responsible. Nevertheless, it is important to acknowledge that the significance thresholds we used in the RNA-seq data analysis were highly stringent. In future, conducting analogous experiments on additional cBCL cell lines will be crucial for evaluating the biological and molecular impacts of the two drugs examined in this study.

Combining BI2536 and MZ1 led to a greater decrease in cell viability compared to their individual use. These results imply that the anti-tumor efficacy of BI2536 and MZ1 might be partially attributed to their impact on c-Myc expression and subsequent effects on the cell cycle, as suggested by RNA-seq data [11].

cBCL, especially focusing on cDLBCL, represents an excellent animal model mirroring its human counterpart, exhibiting numerous pathological, molecular, and clinical resemblances [32,33]. Incorporating a canine model into the drug development process is increasingly recognized as a promising strategy to address various challenges associated with in vitro and murine models [34]. Our findings indicate promising therapeutic implications for human BCL as well.

## 5. Conclusions

In conclusion, our study offers valuable insights into the mechanisms of action of BI2536 and MZ1 in cBCL and highlights their potential as targeted therapies for this cancer subtype. Further investigation is warranted to elucidate the precise molecular mechanisms underlying their anti-tumor activity and to evaluate their effectiveness and safety in both preclinical and clinical settings.

## Figures and Tables

**Table 1:** Scheme of drugs (BI2536 and MZ1) combinations.

	BI2536	MZ1
Combo 1	2.5nM	20nM
Combo 2	2.5nM	50nM
Combo 3	2.5nM	100nM
Combo 4	7.5nM	20nM
Combo 5	7.5nM	50nM
Combo 6	7.5nM	100nM
Combo 7	15nM	20nM
Combo 8	15nM	50nM
Combo 9	15nM	100nM

## Data Availability

Raw Illumina sequencing data are deposited in the SRA database (GenBank) under accession number PRJNA1099474.

## Supplementary Materials

The following supporting information can be downloaded at: https://www.mdpi.com/article/10.3390/ani14101466/s1, Table S1: Cell viability 2-way ANOVA results; Table S2: Differential expression analysis in CLBL-1; Table S3: Differential expression analysis in KLR-1201; Table S4: GSEA in CLBL-1; Table S5: GSEA in KLR-1201.
